# Implantable Biosensors for Real-time Strain and Pressure Monitoring

**DOI:** 10.3390/s8106396

**Published:** 2008-10-15

**Authors:** Ee Lim Tan, Brandon D. Pereles, Brock Horton, Ranyuan Shao, Mohammed Zourob, Keat Ghee Ong

**Affiliations:** 1 Department of Biomedical Engineering, Michigan Technological University, Houghton, MI 49931, USA; 2 Norinse Technologies LLC, Houghton, MI 49931, USA; 3 Biophage Pharma Inc, 6100 Royalmount, Montreal, QC, H4P 2R2, Canada; E-mail: m.zourob@biophagepharma.net

**Keywords:** magnetic harmonic fields, wireless sensors, passive detection, stress, pressure

## Abstract

Implantable biosensors were developed for real-time monitoring of pressure and strain in the human body. The sensors, which are wireless and passive, consisted of a soft magnetic material and a permanent magnet. When exposed to a low frequency AC magnetic field, the soft magnetic material generated secondary magnetic fields that also included the higher-order harmonic modes. Parameters of interest were determined by measuring the changes in the pattern of these higher-order harmonic fields, which was achieved by changing the intensity of a DC magnetic field generated by a permanent magnet. The DC magnetic field, or the biasing field, was altered by changing the separation distance between the soft magnetic material and the permanent magnet. For pressure monitoring, the permanent magnet was placed on the membrane of an airtight chamber. Changes in the ambient pressure deflected the membrane, altering the separation distance between the two magnetic elements and thus the higher-order harmonic fields. Similarly, the soft magnetic material and the permanent magnet were separated by a flexible substrate in the stress/strain sensor. Compressive and tensile forces flexed the substrate, changing the separation distance between the two elements and the higher-order harmonic fields. In the current study, both stress/strain and pressure sensors were fabricated and characterized. Good stability, linearity and repeatability of the sensors were demonstrated. This passive and wireless sensor technology may be useful for long term detection of physical quantities within the human body as a part of treatment assessment, disease diagnosis, or detection of biomedical implant failures.

## 1. Introduction

Physical parameters such as pressure, stress, strain and fluid flow within the human body are critical indicators for detection of many diseases [1]. Today, many physical parameters in the human body are measured indirectly via *ex vivo* methods such as sphygmomanometry and ultrasound techniques. Although simple and noninvasive, the use of *ex vivo* techniques to predict *in vivo* mechanical mechanisms often fails to deliver reliable and localized analytical results [2]. A technique to obtain accurate results is to insert the sensor into the human body via catheter and to perform *in vivo* measurements. For example, the measurement of biliary pressure, which is critical to diagnose sphincter of Oddi (SO) dysfunction, is currently conducted with SO manometry (SOM) [3] that uses a catheter-tip pressure sensor. The major limitation of using the catheter-based measurement technique is that it often requires the patient to be sedated, which may not reflect the actual conditions during the patient's daily activities. This may result in poor prognostic and diagnostic outcomes.

An alternative technique to perform *in vivo* measurements without disrupting the patient's normal activities is the use of implantable biosensors. The sensor is first implanted at the site of interest, with data collected and remotely transmitted to an external recorder in real time. Upon completion of the measurement, which can be as short as a few hours or as long as a few years, the sensor is then removed. The advantages of implantable biosensors are that they can directly track the parameters of interest at the local sites without interrupting the patient's normal activities. The use of implantable biosensors may also reduce the chance of developing infections since there is no need for wires perturbing from inside the body for data collection.

Although their popularity has increased, implantable biosensors are relatively new technologies and are mostly used for orthopaedic applications with stress/strain monitoring. For example, implantable strain sensors based on thin-film metal strain gauges embedded in a polydimethyl-siloxane (PDMS) membrane were used to assess bone morphology [4]. The implantable strain sensor was used to investigate bone diseases such as osteoporosis and bone tumor [5]. A wireless, strain sensor was also developed by MicroStrain Inc. to report real-time forces and torques of a human knee implant. The obtained data served as a guide towards future implant design and as an indicator of the condition of knee implant in order to prevent implant failures.

Implantable biosensors have also been applied for monitoring of cardiovascular systems. An intravascular pressure sensor, consisting of a telemetric pressure sensor inside a silicone capsule [6], was developed to retrieve real-time pressure conditions. The data and power transmission of this sensor were based on the load modulation and inductive coupling techniques, respectively. It was shown that under static pressure condition, the sensor signal was linearly correlated with the applied pressure. Similarly, implantable pressure sensors have been used to detect endoleak and enlargement of an endovascular aneurysm repair (EVAR) on an abdominal aortic aneurysm (AAA) [7]. The sensor consisted of an ultrasound-activated remote pressure transducer that was hand-sewn at the outer surface of the stent graft for direct exposure to aneurysm sac pressurization. The pressure transducer transmitted its signal via ultrasonic waves.

### 1.1. Operating Principle

**Figure 1.**
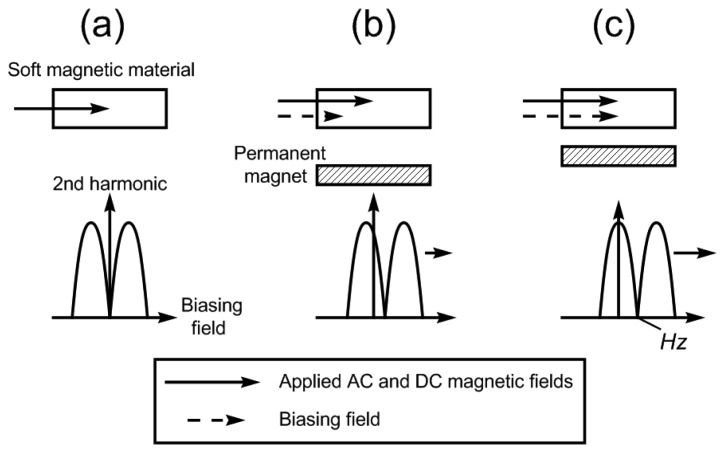
The operational principle of the described implantable sensor. (a) In the absence of the permanent magnet, the soft magnetic material generated a symmetrical 2^nd^ order harmonic field as a function of the applied DC field. (b) The placement of a permanent magnet, which created a secondary DC field (biasing field), shifted the harmonic field pattern. (c) As the separation distance between the two magnetic elements decreased, the secondary biasing field experienced by the soft magnetic material increased. This further increased the shift of the harmonic field pattern.

In this study, an implantable stress/strain sensor and a pressure biosensor were developed and characterized. The sensor, which was wireless and passive, consisted of a soft magnetic material and a permanent magnet [8-11]. As illustrated in Figure 1, the soft magnetic material, referred to as the sensing element, generated secondary magnetic fields that also included the higher-order harmonic modes when it was exposed to a low frequency applied AC magnetic field.

**Figure 2.**
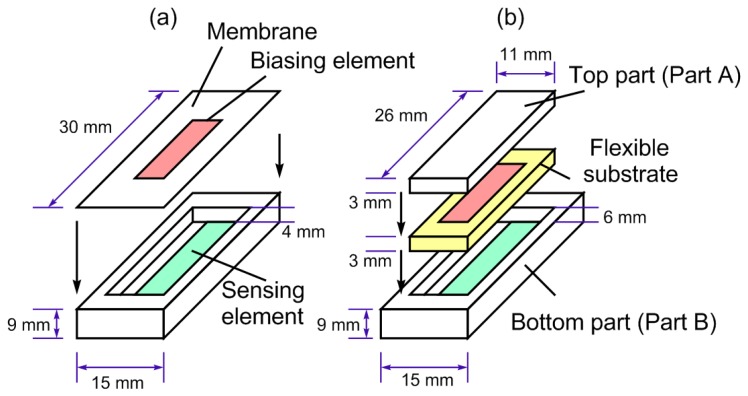
(a) The design of the magnetic harmonic pressure sensor. The biasing element was embedded on a flexible membrane and the sensing element was on a rigid substrate. (b) The design of the magnetic harmonic strain/stress sensor. The biasing and sensing elements were separated by a flexible substrate, and the whole structure was encased within two plastic parts.

The pattern of the higher-order harmonic fields was determined by measuring the sensor's field intensity as a function of an applied DC magnetic field. In this work, the 2^nd^ order was measured due to its larger field intensity compared to other higher-order modes. In the absence of a secondary DC magnetic field (besides the applied DC field), the 2^nd^ harmonic field pattern is symmetrical and has a null at zero biasing fields as shown in Figure 1a. In the presence of a permanent magnet, the 2^nd^ order harmonic field pattern shifted horizontally due to the introduction of the DC magnetic field (biasing field) generated by the permanent magnet, or the biasing element. To quantify the shift, a variable, *H_z_*, was defined as the null point between the two peaks of the 2^nd^ harmonic (see Figure 1). The pressure and stress/strain sensors were designed so the separation distance between the soft magnetic material and the permanent magnet would vary with the parameter of interest. As the separation distance between the two magnetic elements decreased, the biasing field experienced by the soft magnetic material increased. This further increased the shift (*H_z_*) in the harmonic field pattern, allowing remote tracking of the parameter of interest. Figure 2 illustrates the design of the pressure and stress/strain sensors. For the pressure sensor, the biasing element was placed at the membranes of an airtight chamber. Changes in the ambient pressure deflected the membrane, altering the separation distance between the two magnetic elements and thus the higher-order harmonic fields. Similarly, the biasing and sensing elements were separated by a flexible substrate in the stress/strain sensor. Compressive and tensile forces flexed the substrate, changing the separation distance between the two elements and hence the higher-order harmonic fields.

The described sensor technology may have advantages in certain biomedical applications compared to other implantable physical biosensors. The wireless and passive natures of the presented sensor allow long-term implantation in human bodies without worrying about battery lifetime issues. In addition, the sensor is simple in design compared to wireless, passive sensors that are based on microelectronic circuits such as those based on the radio frequency identification technology [12,13]. The shift in higher-order harmonic field is solely a function of the DC biasing field and is independent to mass loading and viscous damping force. Therefore, unlike wireless passive sensors based on the vibrating magnetoelastic sensor technology, the harmonic sensor can be installed inside a human body without affecting the sensor performance. Compared to other magnetic harmonic sensors that detect pressure/stress based on the changes in its harmonic field amplitude [8-11], the described sensor technology will have the advantage of tunable stress/pressure sensitivity by changing the elasticity of the membrane or the flexible substrate.

## 2. Experiments

### 2.1. Fabrication of the Sensing and Biasing Elements

The sensing element was made of a magnetically soft material, Metglas 2826MB (Fe_40_Ni_38_Mo_4_B_18_), purchased from Metglas Inc (Conway, SC, USA) that could generate a large higher-order harmonic field upon excitation by a low-frequency magnetic field. This material has negligible hysteresis in its magnetic BH response and a large and linear initial permeability, thus capable of generating a large magnetic flux in response to an excitation field. Conversely, the permanent magnet was made of Arnokrome III (Arnold Magnetic Technologies, Marengo, IL) strip. This material retained its magnetization state in the absence of a magnetic drive field, thus generating a constant magnetic field. Its large coercive force of 16 kA/m also prevented accidental re-magnetization by stray magnetic fields. Both materials were purchased from the suppliers in rolls, and they were mechanically sheared into desired dimensions.

### 2.2 Pressure Sensor Fabrication

The pressure sensor, illustrated in Figure 2a, was carved from a polycarbonate block with a CNC milling machine. The dimensions of the sensor were 30 mm × 15 mm × 9 mm, and the well was 26 mm × 11 mm × 4 mm. The sensing element was applied to the rigid bottom of the well. The flexible membrane, made of a Mylar sheet of 10 μm, was glued onto the well to form an airtight seal. The sensor was essentially a gauge pressure sensor calibrated at sea level pressure (a reading of 0 Pa was actually 101.325 kPa in absolute pressure). The dimensions of the sensing element were 25 mm × 6.5 mm × 26 μm, and the dimensions of the biasing element were 20 mm × 2.5 mm × 30 μm.

### 2.3 Strain/stress Sensor Fabrication

As illustrated in Figure 2b, the sensing element and biasing element were separately embedded in the top and bottom parts of the polycarbonate sensor package. The dimensions of Part A and B were 26 mm × 11 mm × 3 and 30 mm × 15 mm × 9 mm, respectively. Part B also had a well that was 11 mm × 26 mm × 6 mm in size. Shallow grooves of 0.5 mm were carved in both parts at sides facing the flexible substrate to allow both elements to reside within. The groove in Parts A and B were both measured at 26 mm × 7 mm × 0.5 mm. Deformable rubber pads with tensile strength of 0.66 MPa and 6.21 MPa were used to separate these two elements. The contacting surface of Part A and Part B to the rubber was covered with a thin polycarbonate sheet of 26 mm × 11 mm × 1 mm to ensure uniform force distribution. Part A and Part B were hermitically sealed with silicone glue. The dimensions of the sensing element were 25 mm × 6.5 mm × 26 μm, and the dimensions of the biasing element were 20 mm × 2.5 mm × 30 μm.

### 2.4. Experimental Setup

**Figure 3.**
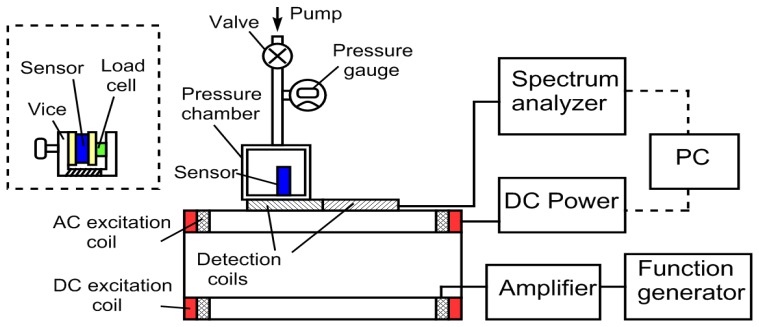
The experimental setup showing the remote interrogation of the pressure sensor. The cutoff on the left side of the figure shows the setup for stress/strain measurements. Dashed lines indicate controlling cables among the PC and other instruments.

Figure 3 illustrates the experimental setup. The excitation coil consisted of two sets of superimposed Helmholtz coils: an AC coil and a DC coil. Each set of coils was 25 cm in diameter, and made of 50 turns of 18-gauge laminated copper wire on each side. The AC coil was connected to an AC function generator (Fluke 271 10MHz) and an amplifier (Tapco J1400), and the DC coil was connected to a computer-controllable DC power supply (Kepco MBT 36-10M). The detection coil was made of two oppositely wound square coils (each coil was 10 cm × 6 cm, 200 turns of 36 gauge laminated copper wire). As illustrated in the figure, an Agilent spectrum/network analyzer 4396A was used to process the captured sensor response. A PC controlled the experiment with the instruments through a GPIB interface using customized software programmed with Visual Basic. To determine the shift in the 2^nd^ harmonic field spectrum, the AC field frequency was set at 200 Hz, and the DC field was varied from 0 to 140 A/m to determine *H_z_*.

For pressure measurements, the sensor was placed inside a pressure chamber, which was placed on the detection coil. The static pressure inside the chamber was measured with a digital absolute pressure gage, and the chamber was pressurized with a bicycle pump. On the other hand, a vice was used to provide the desired stress on the stress/strain sensor. The stress on the sensor was measured with a digital load cell, and the strain was measured with a digital caliper.

## 3. Results and Discussions

### 3.1. Sensor Characterization

**Figure 4.**
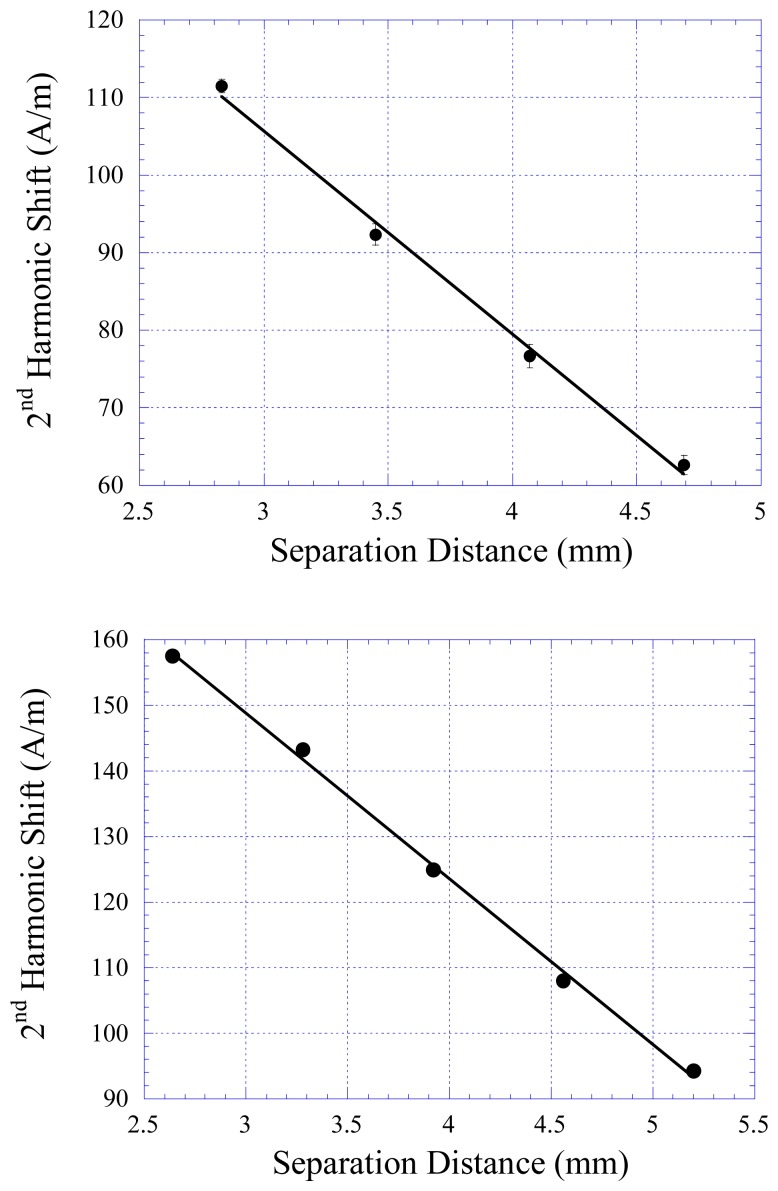
The response of the 2^nd^ harmonic signal of a) the pressure sensor and b) the stress/strain sensor with respect to the separation distance between the sensing and biasing elements.

The 2^nd^ order harmonic field shifts of the pressure and stress/strain sensors were measured as a function of the separation distance between the biasing and sensing elements, and the results are plotted in Figure 4, where Figure 4a presents the response of the pressure sensor and Figure 4b shows the response of the stress/strain sensor. The decreasing *H_z_* with increasing distance was expected since the magnetic harmonic shift was dependent on the strength of the biasing field from the permanent magnetic element. Although the DC biasing field decrease was not linear with distance, the harmonic response of the sensor showed a linear response due to the small change in separation distance.

### 3.2. Pressure Measurements

**Figure 5.**
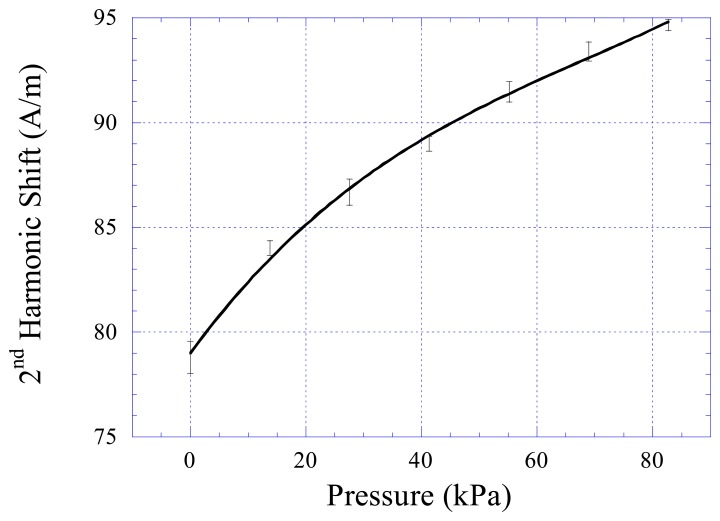
The 2^nd^ order harmonic shift of the sensors at varying pressures from 0 to 83 kPa (12 psi).

Although pressures vary in different parts of a human body, most of them are under 10 kPa. The highest pressure in a human body is the aortic pressure, where in extreme cases it can reach to 300 mmHg (40 kPa). To cover all potential biomedical applications, the pressure range used in the experiment was set from 0 to 83 kPa. The pressure sensor was exposed to varying pressures from 0 to 83 kPa (12 psi), and the shift in harmonic spectrum, *H_z_*, was recorded. Figure 5 plots the responses of the sensors, determined by averaging four measurements at each pressure setting. The harmonic shift of the sensor increased with increasing pressure, and the sensor response followed a polynomial profile.

**Figure 6.**
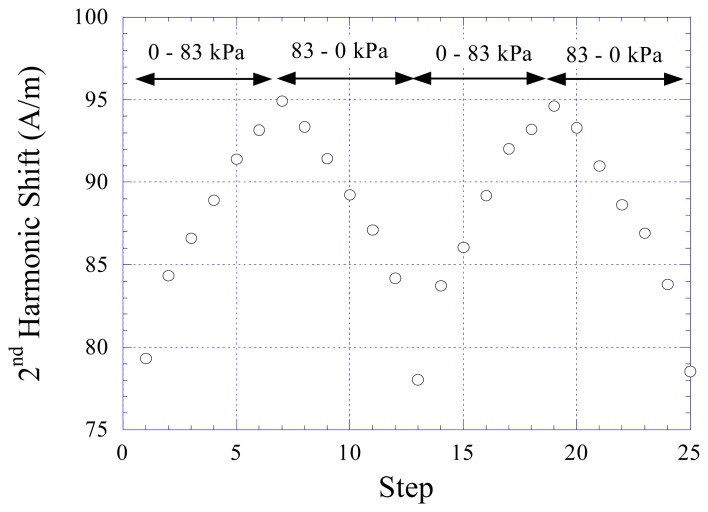
The response of the pressure sensor at increasing and decreasing pressure cycles showed the sensor was repeatable and had no drift.

By correlating the results in Figure 5 to the calibration data in Figure 4a, it was found that the deflection of the membrane was about 500 μm from 0 pressure to 83 kPa. The sensor error, defined as the maximum difference among the measurements at each pressure setting divided by the full-scale output of the sensor, was found to be less than 5%. The repeatability of the pressure sensor was also investigated. Figure 6 shows the response of the sensor at increasing and decreasing pressure cycles. There was no drift for this sensor, and the hysteresis was less than 5%.

### 3.3. Stress/strain Measurements

**Figure 7.**
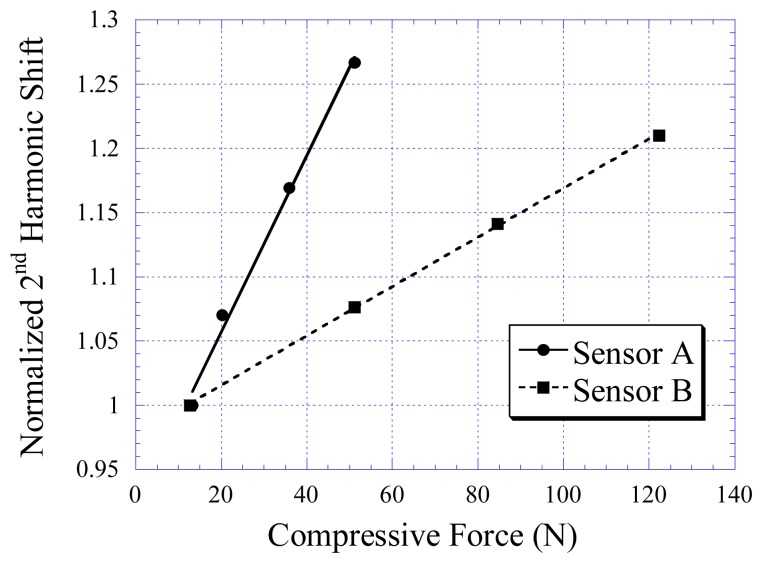
The relationship of compressive stress and changes in the 2^nd^ harmonic shift exhibited by Sensor A and Sensor B.

**Figure 8.**
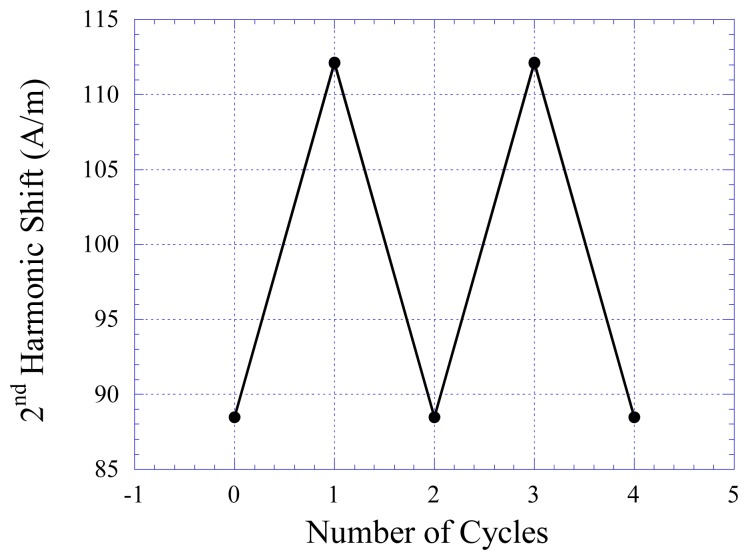
The response of Sensor A is repeatable and stable with cyclic stress variations. The repeatability of Sensor B is similar to Sensor A.

Figure 7 shows the harmonic field shift of the stress/strain sensors as a function of the applied force. Two sensors were tested: Sensor A and Sensor B. The flexible substrate of Sensor A was made with soft rubber material with elasticity of 0.16 MPa, while the flexible substrate of Sensor B was made with harder rubber with elasticity of 2.48 MPa. As shown, the applied compressive force is linearly proportional to the harmonic shift H_z_. As shown in the plot, the harder the rubber material, the lower the sensor sensitivity but the larger the dynamic range. The drift of the stress/strain sensors was also investigated. As shown in Figure 8, there was no visible drift in the sensor response.

## 4. Conclusions

This paper presented the design and fabrication of a novel wireless, passive stress/strain sensor and a pressure sensor for biomedical applications. The sensor tracked the variations in the parameters of interest by measuring the changes in the magnetic 2^nd^ order harmonic field. By investigating the sensor response between 0 and 83 kPa (12 psi) static pressure, the pressure sensor showed a linear response with no drift. For the stress/strain sensors, two types of flexible substrates showed that sensors of higher dynamic ranges (but lower sensitivity) could be built with a stiffer flexible substrate.

Although this paper has demonstrated the design and fabrication of the sensor, several issues such as biocompatibility, long term reliability still need to be resolved before clinical applications. The sensors were fabricated from industrial grade polymers. Thus, in the next phase, equivalent medical grade polymers, such as medical grade polyethylene and Teflon, will be used. The replacement of medical grade materials will not impact the functionality of the sensor as they have similar mechanical properties, although they are more expensive. The current sensor size is still too big for some biomedical applications such as *in vivo* pressure monitoring inside a cardiovascular stent. Therefore, future work such as the miniaturization of the sensor and improving the detection ranges will be conducted to address this issue.
